# Green Chemistry in the Extraction of Natural Dyes from Colored Food Waste, for Dyeing Protein Textile Materials

**DOI:** 10.3390/polym13223867

**Published:** 2021-11-09

**Authors:** Vasilica Popescu, Alexandra Cristina Blaga, Melinda Pruneanu, Irina Niculina Cristian, Marius Pîslaru, Andrei Popescu, Vlad Rotaru, Igor Crețescu, Dan Cașcaval

**Affiliations:** 1 Department of Chemical Engineering in Textiles and Leather, Faculty of Industrial Design and Business Management, “Gheorghe Asachi” Technical University of Iasi, 700050 Iasi, Romania; melinda.pruneanu@academic.tuiasi.ro (M.P.); rotaruvlad1980@gmail.com (V.R.); 2 Department of Organic, Biochemical and Food Engineering, “Cristofor Simionescu” Faculty of Chemical Engineering and Environmental Protection, “Gheorghe Asachi” Technical University of Iasi, 700050 Iasi, Romania; alexandra-cristina.blaga@academic.tuiasi.ro (A.C.B.); dan.cascaval@academic.tuiasi.ro (D.C.); 3 Design and Engineering of Textile Products, Faculty of Industrial Design and Business Management, “Gheorghe Asachi” Technical University of Iasi, 700050 Iasi, Romania; irina-niculina.cristian@academic.tuiasi.ro; 4 Department of Engineering and Management, Faculty of Industrial Design and Business Management, “Gheorghe Asachi” Technical University of Iasi, 700050 Iasi, Romania; marius.pislaru@academic.tuiasi.ro; 5 Department of Machine Design, Mechatronics and Robotics, Faculty of Mechanical Engineering, “Gheorghe Asachi” Technical University of Iasi, 700050 Iasi, Romania; andrei.popescu@academic.tuiasi.ro; 6 Department of Environmental Engineering and Management, “Cristofor Simionescu” Faculty of Chemical Engineering and Environmental Protection, “Gheorghe Asachi” Technical University of Iasi, 700050 Iasi, Romania; igor.cretescu@academic.tuiasi.ro

**Keywords:** betalains, functionalization, esterification, amination, arginine, dyeing, wool

## Abstract

The beetroot peels can be a sustainable source of betalains that can dye the wool materials through green processes based on low water and energy consumption. Green chemistry in the extraction of betalains from colored food waste/peels from red beetroot involved the use of water as a solvent, without other additives. In order for the extract obtained to be able to dye the wool, it was necessary to functionalize betalains or even the wool. Three types of sustainable functionalizations were performed, with (1) acetic acid; (2) ethanol; and (3) arginine. For each functionalization, the mechanism that can justify dyeing the wool in intense colors was elucidated. The characterization of the extract was performed with the data provided by UV-VIS and HPLC-MS analyses. The characterization of the wool dyed with the extract obtained from the red beetroot peels was possible due to the information resulting from the FTIR and CIELab analyses. The functionalizations of betalains and wool in acid environments lead to the most intense red colors. The color varies depending on the pH and the concentration of betalains.

## 1. Introduction

The stringent issue of sustainability and the need for the transition toward a circular economy that are emphasized in EU Directives [1] requires a deep transformation of the textile industry: more and more companies require products to be certified to “STANDARD 100 by OEKO-TEX^®^” [2]. An important direction toward change refers to replacing the synthetic components, especially the synthetic dyes, currently used in the textile industry that are hazardous both to humans and nature. For humans, they can cause allergies, while the dyeing processes lead to polluted residual waters [1]. The solution of this problem is the use of a sustainable approach of design, based on designing or redesigning processes or systems so that their environmental, economic, and societal impact is significantly diminished. It involves novel ways of using biodegradable, non-hazardous raw materials, decreased water and energy consumption, and less pollution/waste [3,4,5].

A biodegradable biomass can be the peels from red beetroot, which is a vegetable widely spread in Romania and in many other places in the world.

Usually, beetroot peels are thrown away even if they contain significant amounts of natural dyes, or at best, they are used for compost. The recovery of these peels by extracting dyes and then using them as nutrients or as natural fertilizer is beneficial and can be a sustainable option [4,5]. Only the component of beetroot pulp has been intensively studied by researchers to highlight its nutritional qualities and health benefits to those who consume it as such in various foods or in the form of juices [4,6,7,8,9].

The dyes extracted from both pulp and red beet peels (betalains) have no affinity for textiles. Betalains (betacyanins, betaxanthins) from beetroot are unstable pigments, which change their color through pH variation or by enzymatic hydrolysis in the presence of light [10,11,12,13,14,15,16]. Prolonged exposure of some beetroot pieces to light, in open spaces, causes their discoloration and browning, which is a phenomenon known to all products containing betalains [10].

However, under certain conditions, betalains could intensely, deeply, and sustainably dye all proteinic textiles.

The main constituent of wool fibers is keratin, a fibrous protein whose polypeptide chains comprise 18 α-amino acids, in well-established relative ratios and sequencing. The particular amino acid in keratin is cysteine, which generates disulfide bridges that crosslink the adjacent polypeptide chains and are responsible for the characteristic properties of keratin: hydrophobicity, water insolubility, high chemical resistance, and low biodegradability [17,18].

The behavior of the keratin macromolecule as an amphoteric polyelectrolyte in aqueous solution is related to the presence of carboxyl (COOH) and amine (NH_2_) side chains. At pH values lower than the isoelectric point, the positively charged protonated amine groups (^+^NH_3_) can interact with anionic species, such as those generated by acid dyes. At solution pH values higher than the isoelectric point, the negatively charged deprotonated carboxyl groups (COO^−^) can interact with cationic species, such as cationic dyes. Wool dyeing with cationic dyes is seldom used; usually, synthetic and natural acid dyes are used, the latter being extracted from different colored sources (vegetables, fruits, plants, insects) [17].

The use of red beetroot peels as a biomass for natural dyes in the textile industry requires knowledge and understanding of the chemical structures of their components, the extraction methods, and their stability depending on pH, temperature, and time [13,14,15,16]. The red color from the beetroot is caused by the presence of the two groups of betalains: betacyanins (red–violet pigments) and betaxanthins (yellow–orange pigments) [6,7,8,9,10,11,12,13,14,15,16]. The types of the dye components identified in the pulp, respectively the peels of the red beetroot (Beta vulgaris), are not so different. Still, the isobetanin component is identified only in the peels. Another significant aspect is the fact that the percentage of components varies significantly—the peels contain up to four to seven times more betanin, which could lead to different tinctorial behavior [11,12,13]. This means that for the same mass, the color strength of the extract from peels is higher than for the extract from pulp; therefore, the extraction process will use less biomass, and the resulting extract will be more concentrated. Furthermore, beetroot pulp has a large range of applications in the food industry, and its use in textile dyeing is not justified.

The literature indicates that red beetroot peels (Beta vulgaris) are richest in betanin, isobetanine, and ferulic acid ester compared to pulp [6,11,12], this leading to the idea of capitalizing on this biomass.

The peels of red beetroot contain the largest amount of total phenolics and betanin, which are distributed in various root parts, decreasing in the order: peel > crown > flesh, while the isobetanin was found in the crown and the peel but not in the pulp. Most varieties of red beetroot contain a large amount of betanin, which can confer 75 to 90% of the total color of betalains. Other betacyanins identified in different parts of red beetroot are isobetanin, prebetanin, neobetanin, betanidin, isobetanidin, amaranthin, lampranthin I, and lampranthin II (feruloylbetanin) [11]. The beetroot peel can contain vulgaxanthin I, vulgaxanthin II, indicaxanthin as betaxanthins and betanin, prebetanin, isobetanin, and neobetanin as betacyanins. In addition, cyclodopa glucoside/V-formylcyclodopa glucoside, glucoside of hydroxyindolcarboxylic acid, betalamic acid, L-tryptophan, p-coumaric acid, ferulic acid, and traces of unidentified flavonoids were detected [12,13].

The dyes’ quantities from the beetroot peels depend on the degree of maturity of the vegetable, variety, and climatic conditions [6].

Extraction of dyes from the pulp of red beetroot (betalains) for the food industry can be done in the presence of water, of an acid/alcohol medium (citric acid solution, ascorbic acid solution, citric acid and ascorbic acid mixture, ethyl alcohol, citric acid and ethyl alcohol mixture, HCl mixture, and ethyl alcohol) or by enzyme/microorganisms-assisted processes [5,6,7,8,9,10].

The stability of betalains is influenced by numerous factors, as follows: pH, temperature, time, dye content, glycosylation degree, acylation degree, antioxidants, chelating agents, and nitrogen atmosphere [13,14,15,16]. Modifying these factors, the color of the dye is often affected.

Studying the literature on the extraction methods of the dyes from the red beetroot with the possibility of their use in the textile industry, we reached the following conclusions: (a) There are a lot of references concerning the extraction of red beetroot dyes (for juices in the food industry) [10,11,12,13,14,15,16], but no such extract was used in the textile industry [5]; (b) The vegetables dyes are currently extracted mostly from the pulp, ignoring the peels, which are often a by-product of the food industry [11,12,13,14]; (c) Nobody has succeeded in a red deep and durable dyeing of wool with functionalized extract from red beetroot peels.

Regarding the dyeing process, the protein materials (wool and silk) are not dyed with fresh red beetroot extract; if a mordant is added to the dye bath, then only light colors such as “milk rose or pink baby” are obtained [19,20].

Certain mordants added to the dyebath (potassium dichromate, aluminum sulfate, ferrous sulfate, copper sulfate) had a negative effect on the color change performance of some natural support (wool and wood) [21,22]. The silk dyeing using simultaneously a mordanting technique, in the presence of ecofriendly acid mordants (citric acid, tartaric acid, acetic acid and tannic acid), led to good color fastness properties and some changes in shade, depending on the mordant used [23].

The research direction of this article has a practical approach and could be useful for solving current global problems related to sustainable production. The capitalization of red beetroot peels and the functionalization methods could open new paths to green dyeing processes without chemicals harmful to the human factor and the environment.

The main objective of this work is to increase the sustainability of textile materials by developing an environmental-friendly innovative method for the dyeing of wool materials using recycled vegetal waste. 

The novelty of the study consists in the intense red coloring of the wool using a sustainable extract obtained from beetroot peels, without using metallic mordants. Three types of functionalization of the extract and respectively of the textile support were performed: (1) functionalization in an acid environment; (2) esterification; and (3) amination. The mechanism that justifies the dyeing of the wool in strong red colors was determined for each type. Using the UV-VIS and HPLC-MS analyses, we determined the component of the extracts. FTIR and CIEL*a*b* measurements confirmed the existence of betalains on the textile support, giving it a red color with certain characteristics.

Color fastness properties are very good in two cases: (1) when the wool has been dyed with an extract functionalized in an acidic environment and (2) when the wool has been functionalized in an acidic environment before dyeing. The designation of one of these variants as the best must be based on the analysis of color in terms of strength, shade, resistance to washing, rubbing, exposure to light, and durability to repeated washings. The destination of the dyed material and the client’s requirements/preferences are also other criteria for selecting the optimal functionalization/dyeing variant.

## 2. Materials and Methods

### 2.1. Materials and Chemicals

The protein materials used were wool in the form of fabric with 250 g/m^2^. To extract the natural dye, beetroot peels were used (from Romania), and 80% acetic acid and 96% ethanol were purchased from Merck and L-arginine from the company Sanflora from Bucharest/Romania. 

### 2.2. Experimental Protocol

For the extraction of betalains, 200 g of beetroot peels were used in 2 L of distilled water, proceeding as follows: after washing the beets, the peel was grated, crushed, weighed, and then added to a 3 L Berzelius glass. Distilled water (2 L) was added (without other additives), and everything was well stirred for 1 h, at room temperature. After filtration, volumes of 100 mL extracts were used for the functionalization of betalains and wool dyeing.

Three types of functionalizations were performed both on the wool samples and betalains/extracts, as follows:

(1) Treatment with 10 mL of acetic acid;
(2) Esterification with 10 mL ethanol in an acid medium (pH = 3);
(3) Amination with 10 mL arginine solution of 50 g/L.
(4) The duration of each functionalization was 30 min, at 25 °C. After the functionalization, the wool samples of 1 g each were dyed, at 40 °C, for 2 h.

**Figure 1:**
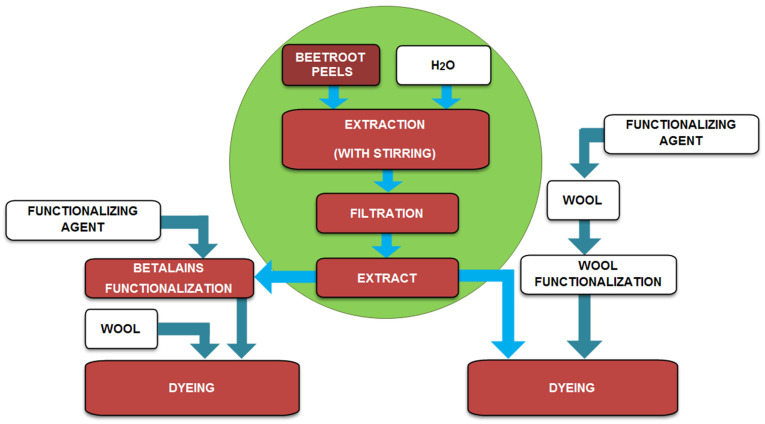
Diagram showing the steps performed for betalains extraction, functionalization treatments, and wool dyeing.

Therefore, the experimental protocol includes a green chemistry extraction of betalains from red beetroot peels, betalains/wool functionalization treatments, and wool dyeing; all these processes are highlighted in Figure 1.

The dyed wool samples were abbreviated according to the functionalization made, as follows:

(a) When the functionalization was performed on wool: (1) FAAW—functionalization with acid acetic; (2) FEW—functionalization with ethanol; (3) FAW—functionalization with arginine.
(b) When the betalains/extract has been functionalized: (1) FAAD—functionalization with acid acetic; (2) FED—functionalization with ethanol; (3) FAD—functionalization with arginine.

The quality of the dyed samples was verified by performing the following color fastness tests:

- ISO 105-C10: 2010—Color fastness to washing test;
- ISO 105-X12—Color fastness to rubbing test (also known as the “Crock test”);
- ISO 105-B02: 2013—Color fastness to light test.

In order to appreciate the results of the washing tests, the 2 variants of the gray scale were used: one for assessing the change in color and another one for assessing staining of the undyed lining fabric. In the case of color fastness to rubbing, only the gray scale to assess the staining was used. The light fastness was assessed with the blue wool scale that has 8 references/swatches.

Durability at repeated washings was tested using the Home Laundering test (SM SR EN ISO 105-CO6: 2013). This testing is based on gravimetric determinations; for the functionalized and dyed samples, the mass losses were measured after 1–5 cycles of repeated washings. The washes were carried out in accordance with the instructions from the Home Laundering test, on Warner Mathis equipment (Switzerland). The gravimetric measurements were performed using an electronic balance of the Mettler Toledo AT201 type that can assess any mass with an accuracy of 0.01 mg.

### 2.3. UV-VIS

The UV–VIS spectra were performed in 200–800 nm range on a Single Beam Scanning M 501 UV–VIS spectrophotometer. This analysis allowed determining the dependence of the absorbance on the extract concentration and drawing a calibration curve. The characterization of the extract was performed according to the reports in the literature [24,25,26,27] using absorbances at different wavelengths and at different pH values. The following parameters were calculated: betanin content, the total monomeric anthocyanins, color density (control), browning index, and percentage of polymeric color.

The relationships used to characterize the extract are as follows:

- Betanin content (mg/g fresh weight) = A × MW × V_f_ × DF/ε × L × W_f_;
- Betanin content (mg/L) = [(A × DF × MW × 1000/ε × L)];
- The total monomeric antocyanins (mg/L) = A × DF × MW × 1000/ε × L;
- Color density (control) = (A_420 nm_ − A_700 nm_) + (A_vis max_ − A_700 nm_);
- Polymeric density (bisulfite-treated sample) = (A_420 nm_ − A_700 nm_) + (A_vis max_ − A_700 nm_);
- Browning index (bisulfite-treated sample) = A_420 nm_ − A_700 nm_;
- Percent of polymeric color = polymeric color density × 100 %/color density.

where:

A is the absorption at λ_max_ corrected by the absorption at 600 nm or even 650 nm; 

MW is the molecular weight of betanin;

V_f_ is the total extract volume;

DF is the dilution factor;

ε is molar absorptivity, ε = 60,000 L/mol*cm in water, for betanin;

L equals path length, L = 1 cm; 

W is the weight of fresh (W_f_) of the peels red beetroot;

A_vis max_, A_700 nm_, and A_420 nm_ are the absorbance of extract at maximum wavelength, 700 nm, and 420 nm at certain pH.

### 2.4. HPLC Experimental Technique

For the identification of the compounds in the beetroot extract, a Thermo HPLC-MS system was used, equipped with a Diode Array and MSQ Plus detector, an Acclaim 120 C18 column (150 mm × 4.6 µm), acetonitrile (5–95%), and formic acid 0.1% (95–5%), with a flow rate of 0.4 mL/min as mobile phase and 10 µL injection volume. The MSQ plus functioned in ESI mode (positive ion mode), with 250 °C probe temperature, and +75 V (cone). The UV absorptions were recorded at 4 wavelengths: 310 nm, 480 nm, 505 nm, and 541 nm.

### 2.5. FTIR

To acquire and record the IR spectra, equipment from Bruker Optic, Germany was used, consisting of an FT-IR spectrophotometer coupled with a HYPERION 1000 microscope. The process was done in reflection mode, and the spectra were superimposed using KnowItAll software.

### 2.6. CIEL*a*b* Measurements

CIEL*a*b* measurements were performed for extracts as well as for dyed/functionalized samples. CIEL*a*b* measurements (L*, a*, b*, C*, h) were performed with a Datacolor Sprectroflash SF300 spectrophotometer. The meanings of the color measurements are as follows: L* is the lightness; a* and b* indicate the positions on the red–green and yellow–blue coordinates; C* is the color saturation; and h is the color hue. The color strength (K/S) was also measured on a Datacolor Sprectroflash SF300 spectrophotometer that uses the light reflectance technique based on the Kubelka–Munk equation.

### 2.7. Statistical Analysis 

All experiments were performed in triplicate, for which the calculation of the standard error of the mean was required. The calculation was performed in Excel, and the size of each one was indicated directly on the figures.

## 3. Results and Discussion

### 3.1. Characterization of the Extract

#### 3.1.1. UV-VIS

In order to dye the textile materials with aqueous extracts of some natural dyes, it is necessary to know the behavior in the UV-VIS field, betalains content/characteristics of the colored extract, and especially what are the betacyanin and betaxanthin components existing in the extract.

**Figure 2:**
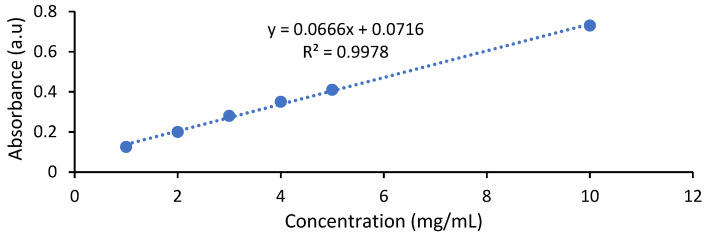
Calibration curve of the extract resulted from peels of red beetroot.

For the raw extract (non-functionalized), a calibration curve was made (Figure 2), which highlights the dependence of the absorption on the concentration of betalains. This calibration curve allows determining the concentrations of betalains from the extracts used for dyeing, which is extremely useful information when it is desired to reproduce the same color on several batches of textiles. The characteristics of the extract resulted from peels of red beetroot are indicated in Table 1, being calculated according to the indications in the literature [24,25,26,27].

The color of this extract (for a volume V = 50 mL, measured at room temperature) is characterized by the following CIEL*a*b* values:

(1) At zero moment, for the mixture of distilled water with beet peels: L* = 3.04, a* = −0.12, b* = −0.12 and C* = 0.12. The color strength is K/S = 2.16;
(2) After 1 h of continuous stirring, the color became stronger and highlighted by the CIEL*a*b* differences from those at time zero: dL* = −0.88, da* = −0.29, db* = 0.39, dC* = 0.03 and color difference dE* = 1.01. The color strength K/S became 2.66.

#### 3.1.2. HPLC 

**Figure 3:**
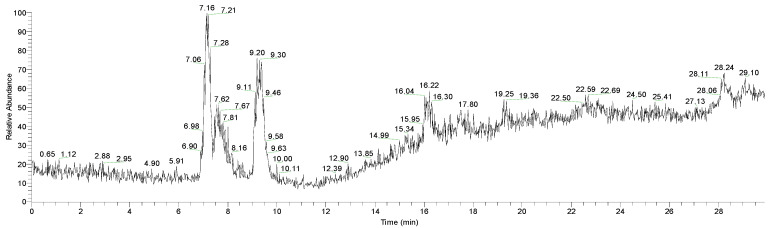

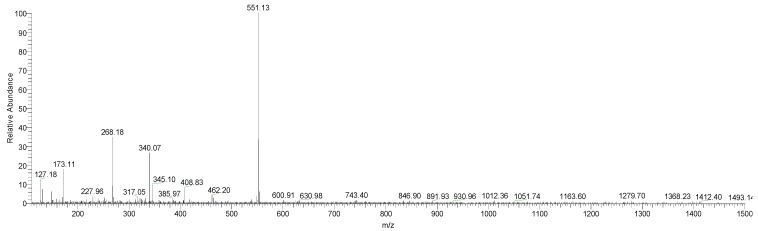
HPLC chromatogram of the betalains compound identified in the red beet peel extract.

At the retention time RT = 9.27, on the mass spectrum of the analyzed extract (containing 142.35 mg/L anthocyanins), the presence of some components of betalains is observed (Figure 3); of these, those that may have a role in dyeing textiles are the following: bethanin (*m/z* = 551), isobetanin (*m/z* = 551), vulgaxanthin I (*m/z* = 340), 17-decarboxy-isobetanidine (*m/z* = 345), and prebetanin (*m/z* = 631). The main component is betanin (*m/z* = 551).

### 3.2. Functionalization and Dyeing of Wool with Red Beetroot Peels Extract

The mechanism of dyeing wool with beetroot extract is based on the following:

1. Functionalization/activation of wool and the formation of ionic bonds with COO-groups in betalains;
2. Functionalization of the dye, making possible the formation of electrostatic interactions between the functional groups of betalains and those of activated wool.

#### 3.2.1. Functionalization of Wool

The three types of functionalization treatments to which the wool was subjected made it possible to dye it in strong red colors. The reaction mechanisms are further presented in detail in Section 3.2.1.1, Section 3.2.1.2, Section 3.2.1.3.

##### 3.2.1.1. Functionalization of Wool in Acidic Environment

The protonation of wool in acidic medium is based on dissociation reactions (of the acid and amino acids in wool), activation of wool, and a double exchange reaction between activated wool and betalains Equations (1)–(4), thus:

(1) $$\begin{array}{c} \;\;\;\;HX\;\;\overset{\;\;\;\;\;\;\;\;\;\;\;}{\to }\;H^{+}+X^{-}\\ acid\;\;\;\;\;\;\;\;\;\;\;\;\;\;\;\;\;\;\;\;\;\;\;\;\;\; \end{array}$$

(2) $$\begin{array}{c} \;\;\;\;\;\;\;\;\;HOOC-Wool-NH_{2}\;\overset{\;\;\;\;\;\;\;\;\;\;\;}{\to }{}^{-}OOC-Wool-{}^{+}NH_{3}\\ \;\;\;\;wool\;\;\;\;\;\;\;\;\;\;\;\;\;\;\;\;\;\;\;\;\;\;\;\;\;\;\;\;\;\;\;\;\;\;\;\;\;\;\;\; \end{array}$$

Wool is an amphoteric compound; therefore, in the acid environment, it interacts with the acid, accepting protons, thus resulting in activated/protonated wool.

(3) $$\begin{array}{c} {}^{-}OOC-Wool-{}^{+}NH_{3}+\;H^{+}+X^{-}\;\overset{\;\;\;\;\;\;\;\;\;\;\;}{\to }{}^{+}H{}^{-}OOC-Wool-{}^{+}NH_{3}X^{-}\\ \;wool\;\;\;\;\;\;\;\;\;\;\;\;\;\;\;\;\;\;\;\;\;\;\;\;\;\;\;\;\;\;acid\;\;\;\;\;\;\;\;\;\;\;\;\;\;\;\;\;\;\;\;actived\;wool\;\;\; \end{array}$$

This intermediate phase actually means an activation of the amino groups of the wool, which thus become binding centers for the anions of the dye in the solution:

(4) $$\begin{array}{c} {}^{+}H{}^{-}OOC-Wool-{}^{+}NH_{3}\;X^{-}+\;Dye-COO^{-}H^{+}\;\overset{\;\;\;\;\;\;\;\;\;\;\;}{\to }\;\;HOOC-Wool-{}^{+}NH_{3}{}^{-}OOC-Dye\;+{}^{+}H{}^{-}X\\ actived\;wool\;\;\;\;\;\;\;\;\;\;\;\;\;\;\;\;\;\;\;\;\;\;\;\;\;\;\;\;\;\;\;\;\;\;\;\;betalains\;\;\;\;\;\;\;\;\;\;\;\;\;\;\;\;\;\;\;\;\;\;\;\;\;\;\;\;\;\;\;\;\;\;\;\;\;\;\;\;\;\;\;dyed\;\;wool\;\;\;\;\;\;\;\;\;\;\;\;\;\;\;\;\;\;\;\;\;\;\;\;\;\;\;\;\;\;\;\;\;\; \end{array}$$

This mechanism is similar to that found in the classic dyeing of wool with synthesized acid dyes that have SO_3_^−^ anionic groups and can form ionic bonds with the ^+^NH_3_ group of wool activated by the acid medium from dyeing. The difference is that betalains acquire COO^−^ anionic groups, only after a deprotonation process, determined by the presence of a weaker acid as betalains (e.g., acetic acid or water).

##### 3.2.1.2. Wool Esterification (FEW) with Ethanol

Wool esterification (FEW) with ethanol, in acid medium (HCl), consisted in the conversion of COOH groups from wool into COO-CH_2_-CH_3_ groups (Equation (5)); as a result, an intense yellow coloration appears on the wool, which combines with the red, determined by the attraction between the free NH_2_ groups, from the wool amino acids, which protonate in the acidic environment (Equation (3)) and form ionic bonds with the COO^−^ groups from betalains (Equation (4)).

If the acid used to carry out the esterification reaction is of the organic type (acetic acid), then the color of the wool is intense red with faint shades of yellow, because the degree of esterification is lower. It turns out that the ^+^NH_3_ groups in wool and COO^−^ in betalains have a significant role in ensuring the red coloration, after dyeing.

(5) $$H_{3}N^{+}-Wool-COOH\;+\;CH_{3}-CH_{2}-OH\;\;\;\overset{+H^{+}}{\to }\;\;H_{3}N^{+}-Wool-COO-CH_{2}-CH_{3\;}+H_{2}O$$

##### 3.2.1.3. Functionalization of Wool with Arginine

**Figure 4:**
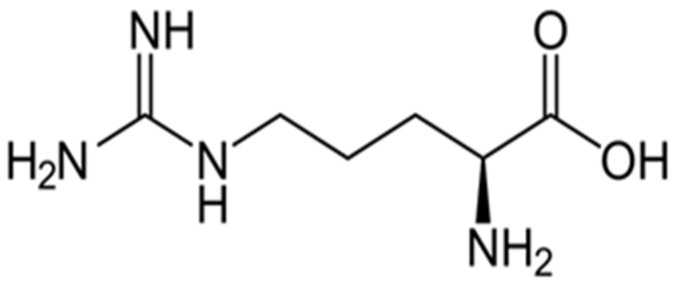
The chemical structure of arginine.

**Figure 5:**
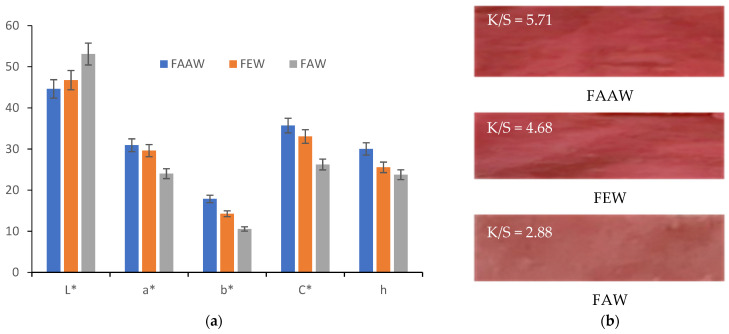
Characterization of the colors resulting from the functionalization and dyeing of the wool: (**a**) CIEL*a*b* measurements; (**b**) color strength (K/S).

The functionalization of wool with arginine determines the enrichment of wool in NH_2_ groups. Arginine is an amino acid that has the chemical structure shown in Figure 4. The newly acquired NH_2_ groups by functionalization with arginine are protonated in the acidic environment used to dye the wool, thus showing attraction for the COO^−^/carboxylic groups in betalains. The red coloration of the arginine functionalized sample (FAW) is much lower in intensity than in the case of FAAW and FEW (Figure 5), which is probably due to the steric hindrance or simultaneous attraction of the dye molecule by the numerous protonated NH_2_ groups from functionalized wool.

The red colors obtained after each functionalization of the wool, followed by dyeing, were evaluated using CIEL*a*b* measurements and color strength, K/S (Figure 5).

Figure 5 shows that the protonated wool (FAAW) with acetic acid led to the strongest red color characterized as follows: L* = 44.62, a* = 30.93, b* = 17.87, C* = 35.72, h = 30.03 and color strength K/S = 5.71.

**Figure 6:**
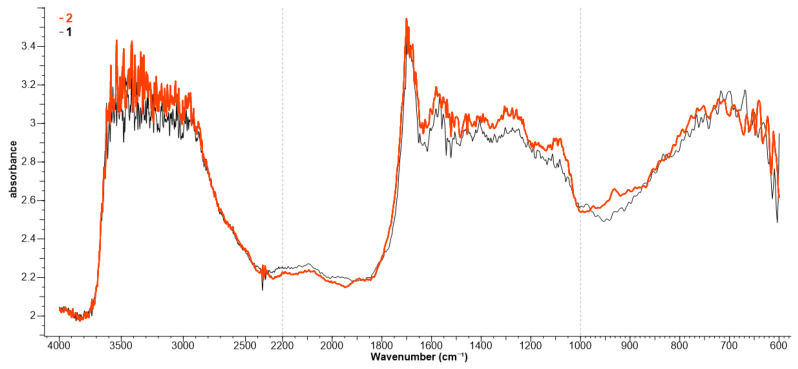
FTIR spectra for: untreated wool (1); protonated and dyed wool (2).

The functionalization/protonation of the wool in acidic environment and its coloring are confirmed by the FTIR analysis (Figure 6). Untreated wool (spectrum 1) records the following absorption bands: 3296 cm^−1^ (N–H and O–H), 2877 cm^−1^ (–CH_2_), 1607 cm^−1^ (Amide I), 1527 cm^−1^ (Amide II), and 1244 cm^−1^ (Amide III). This information is close to that indicated in the literature [28,29,30]. The functionalization of the wool by protonation (spectrum 2) determines changes only at the level of the absorption bands of the free NH_2_ groups that become ^+^NH_3_ (according to Equation (3)). In the untreated wool (spectrum 1), the free NH_2_ groups absorb at 1565 and 1546 cm^−1^; in protonated wool, the ^+^NH_3_ groups absorb at 1581 cm^−1^ (asymmetric deformation) and 1535 cm^−1^ (symmetric deformation). In the range 3200–2800 cm^−1^, the absorptions for ^+^NH_3_ asymmetric and symmetric stretching are observed.

The presence of betalains extract on the dyed samples also determined the slight increase in the following peaks: 3264 cm^−1^ (N-H stretching), 2926 cm^−1^ (C-H stretching), 1627 cm^−1^ (C=O stretching), 1519 cm^−1^ (C=C stretching), 1408 and 1332 cm^−1^ (C-H bending), 1135–1045 cm^−1^ (C-O-C stretching), 1000–750 cm^−1^ (C-H out of plane bending) [31].

#### 3.2.2. Functionalization of Betalains

**Figure 7:**
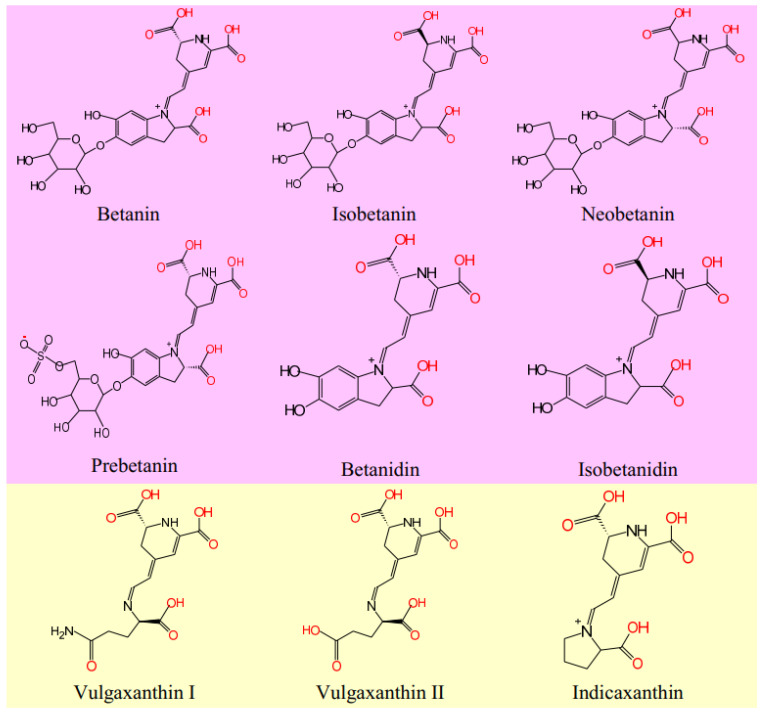
Chemical structure of the main components of betalains: betacyanins and bethaxanthin.

The main components of betalains are betanin, neobetanin, prebetanin, isobetanin, betanidin, isobetanidin, vulgaxanthin I, vulgaxanthin II, and indicaxantihin. The structures of these betalains are indicated in Figure 7.

Without functionalization, the extract does not dye the wool regardless of the temperature used for dyeing and the concentration of the extract. Analyzing the structures of these dyes, it is observed that the functional groups that theoretically could be involved in dyeing wool are of the following types: COOH, OH, NH_2_, and SO_4_^2−^. Their number varies: 3–4 COOH groups, 2–5 OH groups, 1 NH_2_ group, and 1 SO_4_^2−^ group (Table 2).

The presence of polar groups such as COOH, OH, and NH_2_ gives them an affinity for water and a certain solubility, which is why the components of betalains cannot be called pigments but dyes. It is known that pigments are water-insoluble dyes, which can dye any textile material only if they are attached to its surface by means of a binder. In this article, the way in which the aqueous solution of the dyes’ mixture included in betalains can dye the wool materials has been researched. Without a previous functionalization, dyeing the wool with betalains is not possible.

The number of COOH groups in the structure of a betalainic dye gives a certain acid character, which is highlighted by the pKa values. These groups have a significant role in the functionalization of betalains in acidic environments.

##### 3.2.2.1. Functionalization of Betalains in Acidic Environment

**Figure 8:**
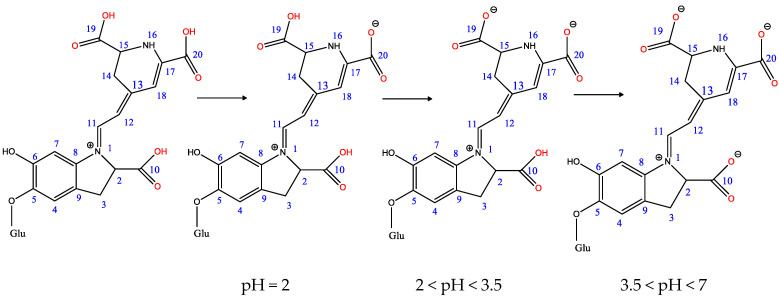
Deprotonation of carboxylic groups according to pH value.

**Figure 9:**
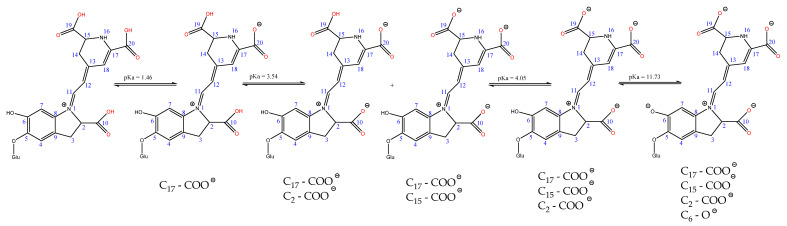
Deprotonation of carboxylic groups in betanin depending on the pKa value.

Acetic acid is added to the colored extract until a pH = 2 is reached; acetic acid is a weaker acid (pKa = 4.75) than betalains (pka = −2.3–2.12, Table 2), the latter will release protons from the COOH groups (Equation (6)), depending on the pH value (Figure 8 and Figure 9). On the other hand, in the acidic environment, the protonation of the NH_2_ groups from vulgaxanthin I (Equation (7)) takes place, which have affinity for the COO^−^ groups from the wool, which they dye in yellow color (Equation (8)). Wool, which contains numerous amino acids, has many COO^−^ and ^+^NH_3_ groups (Equation (2)) as functional groups. The protonated amino groups become dyeing sites. Electrovalent bonds are formed between these groups and COO^−^ from deprotonated betalains (betacyanins and betaxanthin), and the wool will turn red (Equation (9)). Therefore, the final color of the wool dyed in an acid medium, with protonated extract (FAAD) will be yellowish–red, as a result of the combination of the two colors: yellow and red.

(6) $$\begin{array}{c} \;\;\;\;\;\;Dye-COOH\;\;\overset{+CH_{3}COOH}{\to }\;\;Dye-COO^{-}+\;H^{+}\\ \;\;\;\;\;\;\;betalains\;\;\;\;\;\;\;\;\;\;\;\;\;\;\;\;\;\;\;\;\;\;\;\;\;\;\;\;\;deprotonated\;betalains\;\;\; \end{array}$$

(7) $$Vulgaxanthin\;I-NH_{2}\;\;\overset{+H^{+}}{\to }\;\;\;Vulgaxanthin\;I-{}^{+}NH_{3}$$

(8) $$Vulgaxanthin\;I-{}^{+}NH_{3}+{}^{-}OOC-Wool-{}^{+}NH_{3}\;\overset{\;\;\;\;\;\;\;\;\;\;\;}{\to }\;\;Vulgaxanthin\;I-{}^{+}NH_{3}{}^{-}OOC-Wool-{}^{+}NH_{3}$$

(9) $$\begin{array}{c} {}^{-}OOC-Wool-{}^{+}NH_{3}+Dye-COO^{-}\;\;\overset{\;\;\;\;\;\;\;\;\;\;\;}{\to }\;\;{}^{-}OOC-Wool-{}^{+}NH_{3}\;\;{}^{-}OOC-Dye\\ \;\;\;\;\;\;\;\;\;\;\;\;\;\;\;\;\;\;\;\;\;\;\;\;\;\;\;deprotonated\;betalains\;\;\;\;\;\;\;\;\;\;\;\;\;\;\;\;\;\;\;\;\;\;\;\;\;\;\;\;\;\;\;\;\;dyed\;wool \end{array}$$

The realization of Equation (6) depends on the pH value [31], as shown in Figure 8.

The ability of betalains to deprotonate also depends on pKa values; thus, betanin can generate mono-deprotonated, di-deprotonated, and tri-deprotonated forms in the presence of water molecules [32] (Figure 9).

It is observed that deprotonation takes place at the COOH groups level, in the order C17, C17 + C2, C17 + C15, C17 + C15 + C2, and C17 + C15 + C2 + OH6. The degree of deprotonation increases with increasing pH.

In the study [33], the relative stability and bond dissociation energy (thermochemical parameters) of betanin in different deprotonation states has been calculated.

The relative stability decreases in the following order [33]:

(a) In the mono-deprotonated series:

16N^−^ > C17-COO^−^ > C15-COO^−^ > C2-COO^−^ > C6-O^−^; (2 < pH < 3.5);

(b) In the di-deprotonated series:

16N^−^, C6-O^−^ > C17-COO^−^, C6O^−^ > C2-COO^−^, 16N^−^ > C15-COO^−^, C6O^−^ > C2-COO^−^, C17-COO^−^ > C2-COO^−^, C6-O^−^ > C15-COO^−^, C17-COO^−^ > C2-COO^−^, C15-COO^−^ > C15-COO^−^, 16N- > C17-COO^−^, 16N^−^; (3.5 < pH < 7);

(c) In the tri-deprotonated series:

C2-COO^−^, 16N^−^, C6-O^−^ > C2-COO^−^, C17-COO^−^, C6-O^−^ > C2-COO^−^, C15-COO^−^, C6-O^−^ > C17-COO^−^, 16N-, C6O^−^ > C2-COO^−^, C15-COO^−^, C17-COO^−^ > C2-COO^−^, C17-COO^−^, 16N^−^ > C2-COO^−^, C15-COO^−^, 16N^−^; (7 < pH < 9.5).

The deprotonation energies in the mono-deprotonated series are indicated in the same work [33] as follows:

1. 16N^−^ has DE = 256.5 Kcal/mol;
2. C17—COO^−^ has DE = 266.6 Kcal/mol;
3. C15—COO^−^ has DE = 270.00 Kcal/mol;
4. C2—COO^−^ has DE = 270.6 Kcal/mol;
5. C6—O^−^ has DE = 271.9 Kcal/mol.

Even though the nitrogen atom at position 16 can deprotonate most easily, it does not become a dyeing site due to the steric effect that prevents interaction with protonated wool. Probably, the COO^−^ group attached to C17 is responsible for the most intense dyeing.

**Figure 10:**
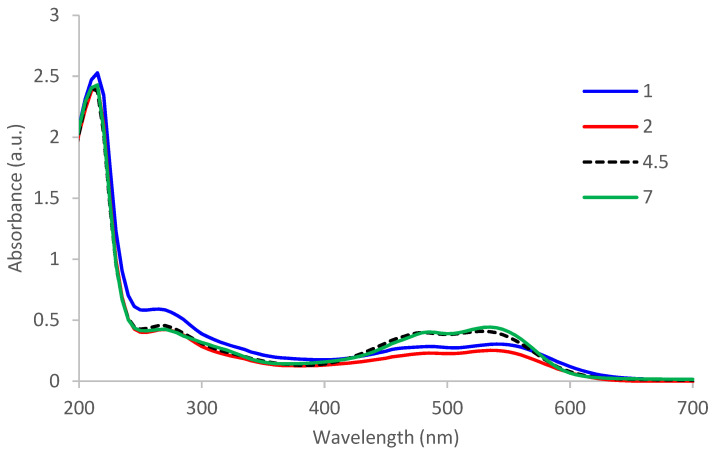
Influence of pH on absorbance of extract/betalains.

The acidic environment influences both the deprotonation process of betalains (Figure 8) and the value of absorption in the UV-VIS domain (Figure 10).

In the case of this extract, blue shift appears, meaning that the hypsochromic effect is probably determined by the changing of solvent’s polarity. The wavelength where the absorption is maximum for betacyanins decreases as the pH increases (λ_max_ = 540.0 nm at pH = 1; λ_max_ = 535.0 nm at pH = 2; λ_max_ = 530.0 nm at pH = 4.5; λ_max_ = 535.0 nm at pH = 7). Maximum absorption decreases as the pH value decreases (hypochromic effect) because at a strong acid pH, there is a degradation of betanin to betanidine (which becomes the main form, including its deprotonated forms) but also the formation of betalamic acid and cyclo-dopa-5-O-glycoside.

The literature [34] indicates that betanidine has maximum absorption at 540 nm, betanin at 535 nm, indicaxanthin at 480 nm, and cyclo-dopa-5-O-glycosides at 270 nm. According to Figure 10, it results that at pH ≤ 2, the aglycons form of betanin is formed, meaning betanidine as result of a hydrolysis process of main betacyanin. Another effect is the increase in absorption from 270 nm, which confirms a certain degradation of betalains in a strongly acidic environment.

The conversion of betanin to a strong acid medium in betanidin has been consistent with other studies in the literature [35,36]. The same effect is obtained at high temperatures or in the presence of a gluco-oxidase (EC 3.2.1.21), and the process is called deglucosylation [35].

The concentration of betanidine is maximum at pH = 1, decreases at pH = 2–5, and then increases to pH = 6–7 [34].

The characterization of the extract functionalized in acidic medium was performed using HPLC analysis. 

The original pH of the betalains extracts in water was pH = 5.7. In the strong acid medium (pH ≤ 2), the betanin can isomerize (to isobetanin or neobetanin) and hydrolyze (to betanidin) [34]. The acid-functionalized extract component, determined by HPLC-MS analysis, is shown in Table 3, compared to the non-functionalized extract component.

It is observed that betanin (551 on the MS spectrum) was converted to betanidine and isobetanidin (*m/z* = 389) when pH = 2 (Table 3).

The results of the HPLC analysis (retention times and identified components of betalains) depend on the content of betalains and the performance of the column/device [12,36,37,38,39].

##### 3.2.2.2. Esterification of Betalains with Ethanol

Esterification of betalains with ethanol (FED) in an acidic medium (pH = 3) depends on the type of acid used [36]: if HCl (pKa = −6) is used, then the esterification effect is high. It means that most COOH groups in betalains esterify [36]; thus, becoming blocked will not attract ^+^NH_3_ groups from the activated wool. In this case, the wool will be dyed in yellow color as a result of the protonation of NH_2_ from vulgaxanthin I and the formation of ionic bonds with the COO^−^ groups from the wool. If acetic acid is used as the acid medium required by the esterification reaction, then the esterification yield is lower, which causes the final color to be red–yellow. It turns out that the COO^−^ groups in betalains have a significant role in dyeing wool, they being the groups that interact with the dyeing sites in activated wool.

##### 3.2.2.3. Functionalization of Betalains with Arginine

**Figure 11:**
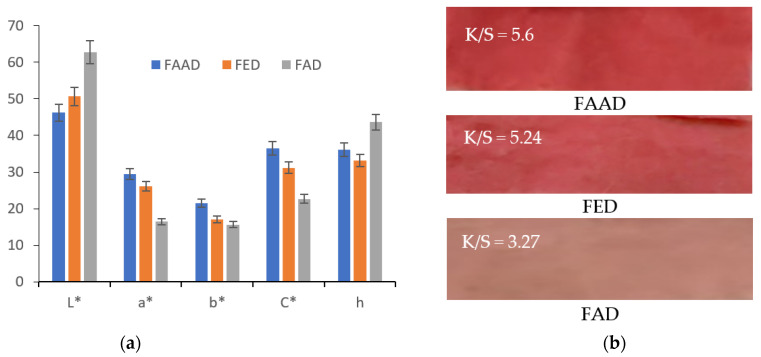
Characterization of the colors resulted after dyeing of the wool with functionalized betalains: (**a**) CIEL*a*b* measurements; (**b**) color strength, K/S.

The functionalization of betalains with arginine (FAD in Figure 11) and then their use in the dyeing of wool in acidic environments causes a weak red color due to the electrostatic attraction between ^+^NH_3_ groups in protonated arginine and the few carboxylic groups left deprotonated (COO^−^) from wool. 

CIEL*a*b* measurements on wool samples dyed with acid-functionalized extract (FAAD) determine the most intense coloration, the reason for which there will still be references only to this type of functionalization. CIEL*a*b* measurements show low values of lightness L* = 46.27 and high values for the others: a* = 29.42, b* = 21.48 and K/S = 5.6 (Figure 11).

**Figure 12:**
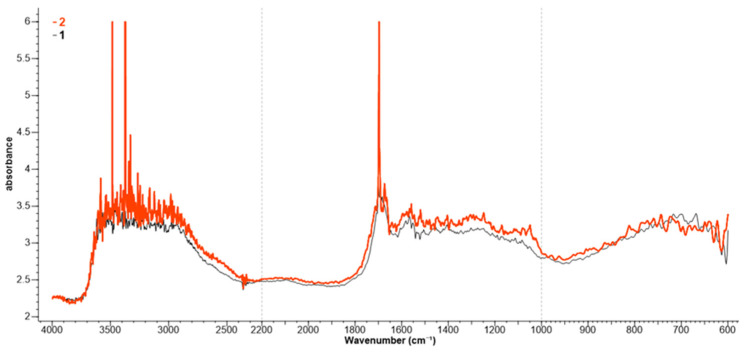
FTIR spectra for: untreated wool (1); wool dyed with protonated betalains (2).

Dyeing the wool with functionalized extract by keeping it in an acidic environment is also confirmed by FTIR analysis (Figure 12).

The functionalization of betalains with an organic acid, acetic acid (pKa = 4.75), determined the deprotonation of some COOH groups (up to COO^−^) and the protonation of NH_2_ groups in Vulgaxanthin I. This behavior is justified by the pKa values of betalains (e.g., pKa for betanin = 1.46) which, in the presence of acetic acid, behave as strong acids, yielding protons. These attach to the free NH_2_ groups in Vulgaxanthin I, converting them to ^+^NH_3_. Thus, zwitterionic forms (H_3_N^+^-R-COO^−^) appear whose presence is proved by the peaks of 1594 cm^−1^ (COO^−^ asymmetric stretching), 1446 cm^−1^ (COO^−^ symmetric stretching), 3102–3000 cm^−1^ (^+^NH_3_ asymmetric stretching), 2985 cm^−1^ (^+^NH_3_ symmetric stretching), 1641 cm^−1^ (^+^NH_3_ asymmetric deformation, weak absorption band), 1558 cm^−1^ (^+^NH_3_ symmetric deformation), and 1023 cm^−1^ (CN stretching). On the other hand, in the case of other betalains (except Vulgaxanthin I), in an acid medium, the dyes exist in the form of R-COO^−^. These COO^−^ ions from betalains interact with the ^+^NH_3_ groups from wool, leading to ionic bonds and a colored wool (Equation (9)).

On spectrum 2, the ^+^NH_3_ groups absorb at 1592 cm^−1^ (asymmetric deformation), 1546 cm^−1^ (symmetric deformation), between 3200 and 2800 cm^−1^ (^+^NH_3_ asymmetric and symmetric stretching), and between 2800 and 2400 cm^−1^ (the weak absorption of ^+^NH_3_ combination bands).

The COO^−^ groups of betalains and COOH of wool absorb at 1697 cm^−1^ (C=O stretching), 1674 cm^−1^ (C=O stretching, internally bonded), 3374 cm^−1^ (dimer OH), and 3482 cm^−1^ (in phenols ArO-H, H bonded) (Figure 12).

### 3.3. The Best Results from Dyeing Wool

Figure 5 and Figure 11 show that the most intense red colors were obtained when acetic acid was used to functionalize wool (FAAW) and betalains (FAD).

#### 3.3.1. Protonated/Activated Wool Dyeing (FAAW)

**Figure 13:**
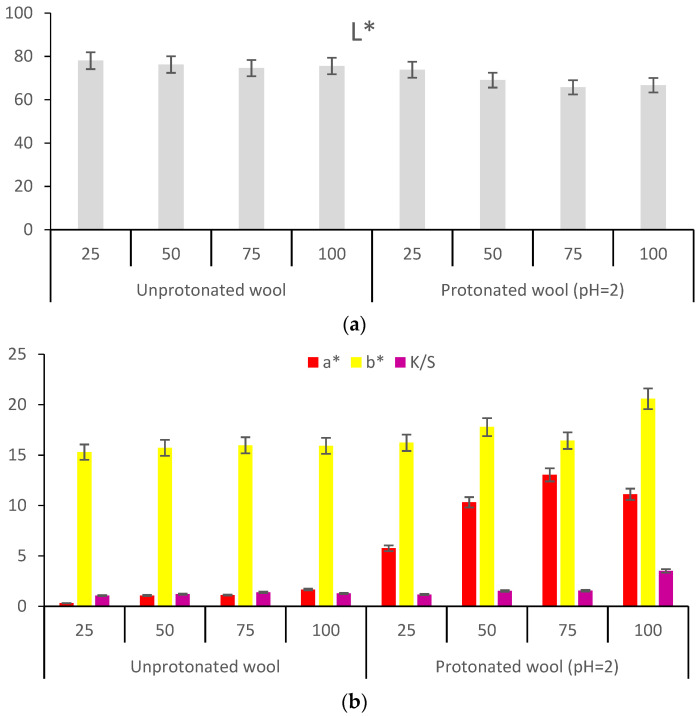
Influence of increasing the volume of colored extract on the color characteristics of wool samples dyed without protonation and with protonation at pH = 2: L* (**a**) and a*, b*, K/S (**b**); dyeing conditions: T = 40 °C, t = 2 h, M = 1:100.

Figure 13a shows that the increase in the volume of extract used to dye the unprotonated wool, at 40 °C, for 2 h leads only to the staining of the wool, the lightness values of L* remaining extremely high (around 78). The decrease in the lightness and the coloring of the wool are very obvious when the wool has been protonated/activated beforehand, at pH = 2. In terms of color, the control sample registers light shades of yellow, and red is almost imperceptible. However, the protonated wool (30 min at pH = 2 with acetic acid) is dyed intensely red, all the more so as the volume of the dyeing solution is higher. This fact is highlighted by both the values of a* and the color strength (K/S = 3.5 in Figure 13b) after a 30 min protonation of the wool and the dye, respectively. 

#### 3.3.2. Wool Dyeing Results with Acid-Functionalized Dye (FAAD)

When the extract has not been protonated, regardless of the volume used for dyeing, the wool does not color because there are no interactions between wool and betalains. Achieving an acidic pH causes the deprotonation of carboxyl groups that can be attracted to ^+^NH_3_ groups in wool. The reddest color is obtained at pH = 3 when a* = 28.13, b* = 12.66, and K/S = 3.0158.

**Figure 14:**
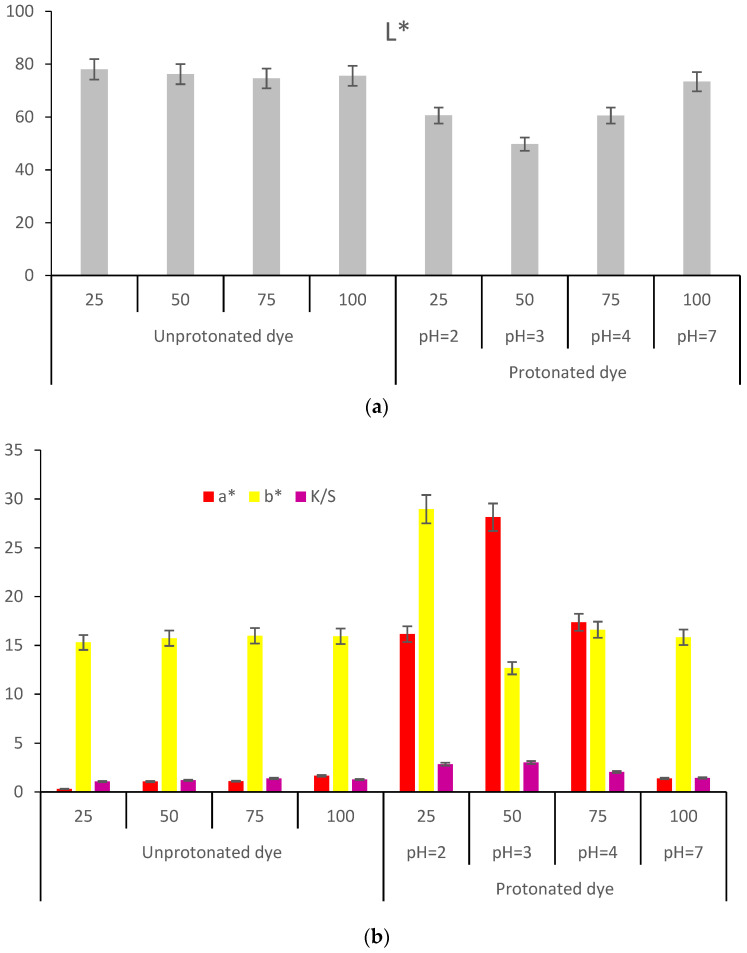
Influence of increasing the volume of colored extract (25–100 mL) on the color characteristics of wool samples dyed with non-protonated extract and with protonated extract at pH = 2–7: L* (**a**) and a*, b*, K/S (**b**); dyeing conditions: T = 40 °C, t = 2 h, liquid ratio M = 1:100.

Dyeing with a large volume of extract (100 mL) with pH = 7 (Figure 14) led to the lowest color strength, which shows that the pH value has a major influence in the process of dyeing the wool.

**Figure 15:**
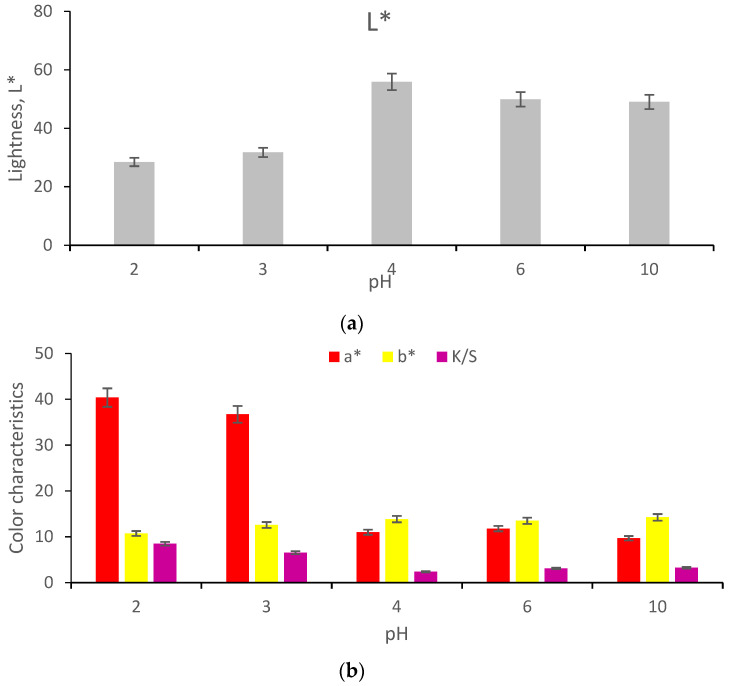
The influence of the pH value on the color characteristics of wool samples dyed with functionalized extract with acetic acid (for acidic pH) and with NH_3_ (for basic environment): L* (**a**) and a*, b*, K/S (**b**); dyeing conditions: T = 40 °C, t = 2 h, V = 100 mL, M = 1: 100.

The use of 100 mL extracts with different pH values led to red colors characterized by lower lightness at lower pHs. Therefore, the higher color strengths and the strongest shades of red were obtained at low pH, which was due to the deprotonation of the extract (Figure 15).

The color variations are generated by the changing ratios of betaxanthin and betacyanin content [34]. The color of the dyed wool samples varies depending on the pH of the dyeing medium, as follows: red-dark at pH ≤ 2, strong red at pH = 3, bright-red at pH 4, and very slow red as the pH = 6.

For an alkaline pH, the color of the dyed wool is yellow–brown because the betalains were degraded by hydrolysis.

**Figure 16:**
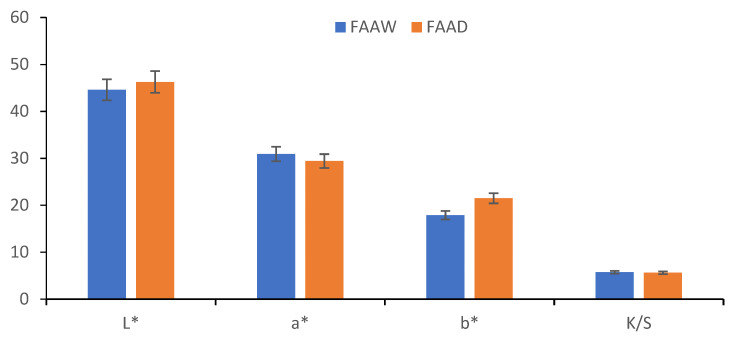
Comparisons between the color characteristics obtained by the functionalizations in acid medium of wool (FAAW) and dye (FAAD).

**Figure 17:**
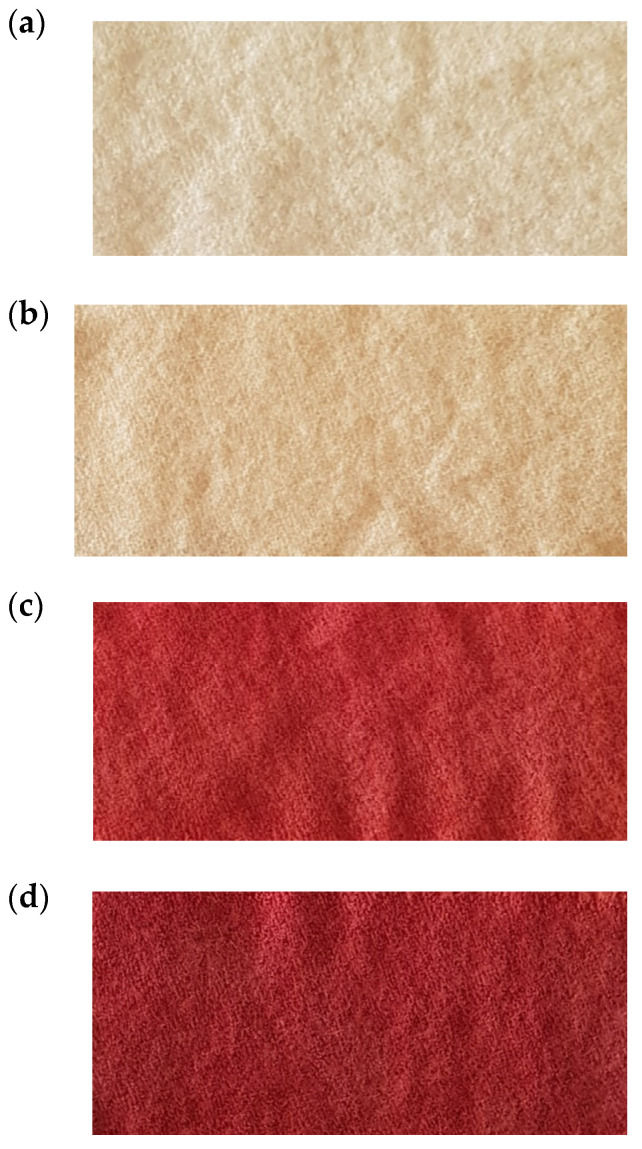
Images of wool samples without and with functionalization, after dyeing: (**a**) untreated wool; (**b**) untreated but dyed wool; (**c**) FAAD sample; (**d**) FAAW sample.

Regarding the shade, Figure 16 and Figure 17 show that FAAD is less red than FAAW, with more yellow, which justifies the effect of the acid: the deprotonation of carboxylic groups but also a slight degradation of betacyanins, with the formation of betalamic acid (yellow). However, the color strengths are extremely close.

#### 3.3.3. Comparison between FAAW and FAAD Samples

The quality of a dyed material depends on the solidity of its shade, which can be verified by performing certain tests. These tests aim to determine the resistance of textile colors to household/commercial washing (when using a reference detergent), to dry or wet rubbing, to light and to repeated household washing. The results of color-fastness tests for samples functionalized in an acidic environment (FAAW and FAAD) are shown in Table 4.

With the exception of light fastness, all the other results obtained are very good, being comparable with the values obtained after the dyeing processes of many natural fibers, performed with synthetic dyes [40,41,42]. This behavior can be explained based on the penetration of betalains inside the wool fiber with which it formed strong bonds.

The ionic bonds are the result of the attraction between the electric charges of opposite sign; charges appeared in deprotonated betalains (COO^−^) and protonated/activated wool (^+^NH_3_), as a result of the functionalizations in an acidic environment. Functionalized betalains behave as high-affinity anionic dyes for the protein support, ensuring very good results at washing and rubbing, such as those included in Table 4.

Color fastness to washing was assessed in terms of changing the color of the dyed sample and staining the two undyed samples, as result of the transfer of dyes from the dyed sample to those undyed, as follows:

- The dyed FAAW sample was assessed with a grade of 5 on the gray scale for assessing the change in color, after performing the color fastness to washing test;
- The undyed cotton and wool samples that were attached on each side of the dyed sample (FAAW and FAAD, respectively) obtained grades of 5 on the gray scale for staining assessment; this indicates that the ionic bonds established between betalains and wool remained intact throughout the washing, even under the action of the detergent and the impact forces exerted by the steel balls used in the color fastness to washing test.

Color fastness to rubbing testing involves evaluating the transfer of dye from the dyed sample to the standard rubbing cloth under dry and wet conditions [41].

The FAAW and FAAD samples have excellent color fastness to dry rubbing (grades of 5) and good values (grades of 4–5) to wet rubbing, on the gray scale for staining assessment.

Regarding the color fastness to light, it is known that natural dyes in general and betalains in particular have a sensitivity to exposure to light [43,44]. The chemical structure of the chromophore is responsible for color loss under the action of light.

The blue wool scale was used to assess the color fading of the dyed tested samples as a result of light action on the chemical structure of dyes.

In Table 4, the values from the light fastness test indicate a certain change in the color of the samples as an effect of light/energy exposure. The light deteriorates the stability of the betalains, leading to the color fading of the tested samples; probably, light affects the chromophores of betaxanthins and betacyanins. However, according to the ISO 105-B02: 2013 standard, grades 4 or 5 on the blue wool scale indicated a fair light fastness, and the betalains will remain unchanged for many years if it is used with additional protection against exposure to light.

The results of the Home Laundering test (SM SR EN ISO 105-CO6: 2013) indicate excellent values for durability because the mass losses after 5 washing cycles are extremely small, representing 1.55% in the case of the FAAW sample and 1.64% in the case of the FAAD sample. In both samples, the ionic bonds between betalains and wool do not break during repeated washings. The small mass losses are probably caused by the dislocation of those dye molecules that failed to get close enough to the dyeing sites to form ionic bonds, but they formed superficial bonds with the protein support.

The comparison of FAAW and FAAD samples must be done taking into account the following aspects:

- In terms of color strength, wool functionalization is better because the FAAW sample has K/S = 5.71 higher than the FAAD sample (K/S = 5.6). The lightness values indicate the same idea: L* = 44.62 in the case of FAAW and L* = 46.27 in the case of FAAD. However, obtaining the FAAW requires increased attention to the handling of the protonated wool, from the functionalization bath to the dyeing bath;
- In terms of shade, the FAAW sample is redder (a* = 30.93) and less yellow (b* = 17.87) than the FAAD sample (a* = 29.42 and b* = 21.48);
- In terms of color-fastness properties, both functionalizations lead to very good results in washing and rubbing and to acceptable results in the light fastness test (Table 4). The values of these tests can be criteria for choosing the type of functionalization, depending on the destination of the dyed fabric;
- In terms of durability for repeated household washes, both functionalizations indicate excellent results.

In conclusion, both variants based on functionalization in an acid environment are good, indicating that the choice of the functionalization process should be made depending on the destination of the dyed material and the requirements/preferences of the client.

## 4. Conclusions

Normally, the red extract obtained from beetroot peels does not generate the red color of the wool because there is no affinity between the two partners in the dye bath; betalains (betacyanins and betaxanthins) and wool contain the same types of functional, non-ionized groups (COOH and NH_2_). The ionization of these groups to COO^−^ and ^+^NH_3_ occurs only during deprotonation and activation processes. Thus, there are attractions between these ionized groups with the formation of electrovalent bonds and the generation of a red coloration of the wool.

The acidic environment is responsible for the phenomena of betalains deprotonation and wool activation; strong inorganic acids do not lead to the intensifications of the red color. Esterification functionalizations, compared to those performed with acetic acid, led to lower color strengths, thus highlighting the essential role of COOH groups during dyeing. Enrichment with amino groups due to amination functionalizations has led to much lighter red shades, which means that NH_2_ groups do not play a major role in the process of dyeing the wool. The only solution that favors the strong red coloration of the wool is the functionalization with acetic acid. The pH value influences both the content of betalains and the color characteristics of the dyed wool.

## Figures and Tables

**Table 1:** Characteristics of extract obtained from peels of red beetroot.

	Characteristics	Value
1	Betanin content (mg/g fresh peels red beetroot)	1.4235
2	Total monomeric antocyanins (betanin content) (mg/L)	142.35
3	Color density	0.597583
4	Polimeric color density (bisulfite)	0.769485
5	Browning index	0.204574
6	Percent of polymeric color (%)	128.76621

**Table 2:** Characteristics of betalains.

Dye Category	Dye Name	Number of Functional Groups				Dissociation Constant		Water Solubility (g/L)
		COOH	OH	NH_2_	SO_4_^2−^	pKa	pKb	
Betacyanins (Red–violet color)	Betanin	3	5	-	-	1.46	−3.6	0.5
	Neobetanin	3	5	-	-	1.43	−3.6	0.18
	Prebetanin	3	4	-	1	−2.3	−3.7	0.85
	Isobetanin	3	5	-	-	1.46	−3.6	0.5
	Betanidin	3	2	-	-	1.55	0.056	0.052
	Isobetanidin	3	2	-	-	1.55	0.056	0.052
Betaxanthin (Yellow–orange color)	Vulgaxanthin I	3	-	1	-	1.64	8.59	0.26
	Vulgaxanthin II	4	-	-	-	1.54	8.58	0.21
	Indicaxanthin	3	-	-	-	2.12	0.082	0.21

**Table 3:** The mass spectral data of betalains from peels of red beetroot.

Sample	Compound	*m/z*	*m/z* (Daughter Ions)
Non-functionalized extract (pH = 5.7) RT = 9.27	Vulgaxanthin I	340	323/277
	17-decarboxy-isobetanidin	345	301.1
	Betanin	551	389/149
	Isobetanin	551	389
	Prebetanin	631	551/389
Functionalized extract at pH = 2; RT = 4.67	Portulacaxanthin III	269	225
	Vulgaxanthin I	340	323/277
	Vulgaxanthin II	341	325/148
	Betanidin	389	345
	Isobetanidin	389	389/345

**Table 4:** Results of color-fastness tests for samples functionalized in an acidic environment (pH = 2).

Sample Code	Color Fastness to Washing			Color Fastness to Rubbing		Color Fastness to Light
	Change in Color	Staining				
		Cotton	Wool	Dry	Wet	
FAAW	5	5	5	5	4–5	5
FAAD	4–5	5	5	5	4–5	4

## Data Availability

Not applicable.

## References

[1] Communication from the Commission to the European Parliament, The Council the European Economic and Social Committee and the Committee of the Regions, Closing the Loop—An EU Action Plan for the Circular Economy. https://eur-lex.europa.eu/legal-content/EN/ALL/?uri=CELEX%3A52015DC0614.

[2] Standard 100 by OEKO-TEX. https://www.oeko-tex.com/en/our-standards/standard-100-by-oeko-tex.

[3] Padma S.V., Dhara S., Padma S.V., Dhara S. (2019). Newer natural dyes for various textiles. New Trends in Natural Dyes for Textiles.

[4] Saxena S., Raja A.S.M., Muthu S.S. (2014). Natural dyes: Sources, chemistry, application and sustainability issues. Roadmap to Sustainable Textiles and Clothing, Textile Science and Clothing Technology.

[5] Yusuf M., Shabbir M., Mohammad F. (2017). Natural colorants: Historical, processing and sustainable prospects. Nat. Prod. Bioprospecting.

[6] Ravichandran K., Saw N.M.M.T., Mohdaly A.A., Gabr A., Kastell A., Riedel H., Zhenzhen C., Knorr D., Smetanska I. (2013). Impact of processing of red beet on betalain content and antioxidant activity. Food Res. Int..

[7] Janiszeska E. (2014). Microencapsulated beetroot juice as a potential source of betalain. Powder Technol..

[8] Bhupinder S., Bahadur S.H. (2014). Chemical composition, functional properties and processing of Beetroot—A review. Int. J. Sci. Eng. Res..

[9] Chhikaraa N., Kushwahaa K., Sharmab P., Gata Y., Panghala A. (2019). Bioactive compounds of beetroot and utilization in food processing industry: A critical review. Food Chem..

[10] Knuthsen P. (1981). Investigations on beetroot colours for the purpose of regulation. Z. Für Lebensm.-Unters. Und Forsch..

[11] Kujala T.S., Loponen J.M., Klika K.D., Pihlaja K. (2000). Phenolics and betacyanins in red beetroot (Beta vulgaris) root:  distribution and effect of cold storage on the content of total phenolics and three individual compounds. J. Agric. Food. Chem..

[12] Kujala T.S., Loponen J.M., Pihlaja K. (2001). Betalains and phenolics in red beetroot (Beta vulgaris) peel extracts: Extraction and characterisation. Z. Naturforsch. C.

[13] Sapers G.M., Hornstein J.S. (1979). Varietal differences in colorant properties and stability of red beet pigments. J. Food Sci..

[14] Herbach K.M., Stintzing F.C., Reinhold C. (2006). Betalain stability and degradation-structural and chromatic aspects. J. Food Sci..

[15] Herbach K.M., Stintzing F.C., Carle R. (2004). Impact of thermal treatment on color and pigment pattern of red beet (*Beta vulgaris* L.) preparations. J. Food Sci..

[16] Antigo J.L.D., Bergamasco R.C., Madrona G.S. (2018). Effect of pH on the stability of red beet extract (Beta vulgaris l.) microcapsules produced by spray drying or freeze drying. Food Sci. Technol..

[17] Rajabinejad H., Bucişcanu I.I., Maier S.S. (2019). Current approaches for raw wool waste management and unconventional valorization: A review. Environ. Eng. Manag. J..

[18] Popescu C., Wortmann F.-J., Müssig J. (2010). Wool–Structure, Mechanical Properties and Technical Products based on Animal Fibres. Structure, Properties and Technical Applications. Industrial Applications of Natural Fibres.

[19] Kumar V., Prabha R. (2018). Extraction and analysis of natural dye. J. Nat. Prod. Plant Resour..

[20] Khalida T., Hage M. (2016). Isolation of natural dye from beet root and its application on wool and thread with different mordants at different temperatures. Int. J. Adv. Eng. Res..

[21] Yeniocak M., Goktas O., Ozen E., Colak M., Ugurlu M. (2018). Determination of leaching features of wood surfaces colored by eco-friendly red beetroot (beta vulgaris) extract. Wood Res..

[22] Yeniocak M., Goktas O., Ozen E., Colak M., Ugurlu M. (2015). Natural Coloration of wood material by red beetroot (beta vulgaris) and determination color stability under UV exposure. Maderas. Cienc. Tecnol..

[23] Yaqub A., Chaudhary N., Bhatti R.A.A., Iqbal Z., Habib-ul-Haq M. (2018). Green extraction and dyeing of silk from Beta vulgaris peel dye with ecofriendly acid mordants. Int. J. Biosci..

[24] Stintzing F.C., Carle R., Socaciu C. (2008). Analysis of betalains. Food Colorants Chemical and Functional Properties.

[25] Popa A., Moldovan B., David L. (2015). Betanin from Red Beet (*Beta vulgaris* L.) Extraction conditions and evaluation of the thermal stability. Rev. Chim..

[26] Neagu C., Barbu V. (2014). Principal component analysis of the factors involved in the extraction of beetroot betalains. J. Agroalimenty Process. Technol..

[27] Sturzoiu A., Stroescu M., Stoica A., Dobre T. (2011). Betanine extraction from beta vulgaris—Experimental research and statistical modeling. U.P.B. Sci. Bull. Series B.

[28] Xu W., Ke G., Wu J., Wang X. (2006). Modification of wool fiber using steam explosion. Eur. Pol. J..

[29] Wang X., Shi Z., Zhao Q., Yun Y. (2021). Study on the Structure and Properties of Biofunctional Keratin from Rabbit Hair. Materials.

[30] Margariti C. (2019). The application of FTIR microspectroscopy in a non-invasive and non-destructive way to the study and conservation of mineralized excavated textiles. Herit. Sci..

[31] Devadiga D., Ahipa T.N., Samanta A.K., Nasser Awwad N., Algarni H.M. (2020). Betanin: A Red-Violet Pigment—Chemistry and Applications. Chemistry and Technology of Natural and Synthetic Dyes and Pigments.

[32] Rodriguez S.A., Baumgartner M.T. (2020). Betanidin pKa Prediction Using DFT Methods. ACS Omega.

[33] Gliszczyńska-Świglo A., Szymusiak H., Malinowska P. (2006). Betanin, the main pigment of red beet-molecular origin of its exceptionally high free radical scavenging activity. Food Addit. Contam..

[34] Dumbravă A., Enache I., Oprea C.I., Georgescu A., Gîrţu M.A. (2012). Toward a more efficient utilisation of betalains as pigments for Dye-Sensitized solar cells. Digest Dig. J. Nanomater. Biostruct..

[35] Pires Goncalves L.C., Martorelli Di Genova B., Augusto Dorr F., Pinto E., Leite Bastos E. (2013). Effect of dielectric microwave heating on the color and antiradical capacity of betanin. J. Food Eng..

[36] Tuwalska D., Starzak K., Szot D., Wybraniec S., Winterhalter P., Jerz G. (2014). Semi-synthesis of red beet betacyanin ethyl-esters by esterification. Challenges Mod. Technol..

[37] Belhadj Slimen I., Najar T., Abderrabba M. (2017). Chemical and Antioxidant Properties of Betalains. J. Agric. Food Chem..

[38] Sawicki T., Bączek N., Wiczkowski W. (2016). Betalain profile, content and antioxidant capacity of red beetroot dependent on the genotype and root part. J. Funct. Foods.

[39] Sawicki T., Juśkiewicz J., Wiczkowski W. (2017). Using the SPE and Micro-HPLC-MS/MS Method for the Analysis of Betalains in Rat Plasma after Red Beet Administration. Molecules.

[40] Fisher A. (2017). Contemporary Wool Dyeing and Finishing, Dyeing Methods for Wool, Training Course-Woolwise.

[41] Memon H., Khatri A., Ali N., Memon S. (2016). Dyeing Recipe Optimization for Eco-Friendly Dyeing and Mechanical Property Analysis of Eco-Friendly Dyed Cotton Fabric: Better Fixation, Strength, and Color Yield by Biodegradable Salts. J. Nat. Fibers.

[42] Dutta S., Bansal P., Wang H., Memon H. (2020). Cotton Fiber and Yarn Dyeing. Cotton Science and Processing Technology. Gene, Ginning, Garment and Green Recycling.

[43] Padma S.V., Dhara S., Padma S.V., Dhara S. (2019). Dyeing Application of Newer Natural Dyes on Cotton Silk and Wool with Fastness Properties, CIE Lab Values, and Shade Card. New Trends in Natural Dyes for Textiles.

[44] Padfield T., Landi S. (1966). The Light-Fastness of the Natural Dyes Source. Stud. Conserv..

